# Resistance to multiple first-line antibiotics among *Escherichia coli* from poultry in Western Algeria

**DOI:** 10.14202/vetworld.2020.290-295

**Published:** 2020-02-15

**Authors:** Meki Boutaiba Benklaouz, Hebib Aggad, Qada Benameur

**Affiliations:** 1 Department of Veterinary Sciences, Veterinary Sciences Institute, Ibn Khaldoun University, Tiaret, Algeria; 2 Laboratory of Hygiene and Animal Pathology, Veterinary Sciences Institute, Ibn Khaldoun University, Tiaret, Algeria; 3 Nursing Department, Faculty of Natural Sciences and Life, Abdelhamid Ibn Badis University, Mostaganem, Algeria

**Keywords:** Algeria, *Escherichia coli*, extended-spectrum β-lactamases, first-line antibiotics, multidrug resistance, poultry

## Abstract

**Background and Aim:**

*Escherichia coli* can cause a number of serious infections both in human and veterinary medicine. Their management is increasingly complicated by the emergence and dissemination of multiresistance to various first-line antimicrobial agents. This study aimed to evaluate the resistance level to the commonly used antibiotics, with a focus on the first-line antimicrobial agents, in *E. coli* strains isolated from poultry in Western Algeria.

**Materials and Methods:**

*E. coli* culture was done on MacConkey agar and their identification was determined by AP20E system. For susceptibility testing, disk diffusion method to 14 antimicrobials, including first-line antibiotics, was used according to Kirby–Bauer disk diffusion method in Mueller-Hinton agar and the results were interpreted according to the Clinical and Laboratory Standards Institute guidelines. *E. coli* isolates were considered as multidrug resistance (MDR) when found resistant to at least one antimicrobial agent of three different families of antibiotics. Double-disk synergy and combination disk tests were used for initial screening and confirmation for extended-spectrum β-lactamases (ESBLs) production, respectively.

**Results:**

A total of 145 *E. coli* strains were isolated in this study. High resistance levels to various antibiotics, including commonly used first-line antimicrobial agents, were recorded in this study. The highest resistance level was observed against nalidixic acid (90.34%, n=131), followed by tetracycline (86.89%, n=126), ampicillin (82.75%, n=120), enrofloxacin (80.68%, n=117) and neomycin (80.68%, n=117), trimethoprim/sulfamethoxazole (73.79%, n=107), norfloxacin (72.41%, n=105) and cephalothin (72.41%, n=105), amoxicillin/clavulanic acid (51.72%, n=75), chloramphenicol (22.75%, n=33), nitrofurantoin (17.24%, n=25), gentamicin (13.10%, n=19), and ceftiofur (3.44%, n=5). Moreover, resistance to multiple first-line antibiotics was also demonstrated in the present study. Overall, 139 out of 145 isolates (95.86%) demonstrated MDR (resistant to at least three antibiotics). In addition, five *E. coli* isolates (3.44%) were confirmed to be ESBL producers.

**Conclusion:**

The alarming rate of *E. coli* resistant to multiple first-line antibiotics in poultry demands intensified surveillance. These results call for taking drastic measures to preserve antibiotic effectiveness and reduce the emergence risks of extensively drug-resistant and pandrug-resistant *E. coli* isolates.

## Introduction

*Escherichia coli* is an important cause of community and nosocomial-acquired infections, especially urinary tract infections, bloodstream infections, surgical site infections, pneumonia, and sepsis [1]. It is also considered as a major pathogen of worldwide importance in commercially produced poultry [2]. These bacteria are mainly responsible for causing colibacillosis, which is economically relevant to poultry producers, as it leads to high mortality and poor egg quality in broilers and laying hen flocks, respectively [3].

Antimicrobials play an important role in animal and human health care. A wide range of antimicrobial agents is used to treat community and hospital infections caused by *E. coli*. Most antimicrobials used for animals are also used in human medicine [4]. However, many antimicrobials used in human medicine are not approved for use in animals. Some of the currently available antimicrobials (fluoroquinolones, aminoglycosides, and third- and fourth-generation cephalosporins) are listed as veterinary critically important antimicrobials and are also considered to be highly important medicines for humans according to the Report of the 22^nd^ meeting of the World Health Organization Expert Committee [5]. Acquired resistance to such antibiotics increases the clinical impact of infections due to *E. coli* and complicates their management in both human and veterinary medicine [6]. *E. coli* infections are of clinical concern because this species is becoming progressively more resistant to currently available antimicrobials [7-9] and especially with the emergence of extended-spectrum β-lactamase (ESBL)-producing isolates. The importance of infections due to multidrug-resistant (MDR) *E. coli* has been increasingly recognized in recent years and they are associated with increased morbidity and mortality [10].

One suspected source of drug-resistant *E. coli* in humans is the use of antimicrobial drugs in agriculture [11]. Irresponsible use of antibiotics in intensive livestock farming is an important factor that can play a crucial role in the selection of antibiotic-resistant bacteria. Indeed, the prevalence of highly antibiotic-resistant *E. coli* was recorded in poultry more frequently than other food-producing animals [12]. Therefore, avian *E. coli* could be considered as an epidemiological indicator for antimicrobial resistance monitoring which becomes necessary to preserve public health [13] and to guide the empirical use of antibiotics [14]. Thus, monitoring *E. coli* for antimicrobial resistance might also provide predictive information on antimicrobial resistance in potentially pathogenic bacteria [15]. A large number of studies have been performed in different parts of the world, focused on the analysis of the prevalence of MDR *E. coli* in poultry [7,12,15]. Such surveillance should be localized and updated as often as possible because resistance trends can vary even between regions in the same city [14], and especially in countries, where antibiotic usage is not strictly regulated.

This study aimed to evaluate the levels of resistance to the commonly used antibiotics, with a focus on first-line antimicrobial agents, in *E. coli* isolates from poultry and to investigate the presence of ESBL-producing isolates.

## Materials and Methods

### Ethical approval

No ethical approval was needed to realize this work.

### Study area

This work was carried out in several regions located in the west of Algeria: Chlef, Mostaganem, Relizane, Tiaret, Mascara, and Tissemsilt.

### Study period and sampling

From January 2017 and March 2019, a total of 290 samples were received to the Laboratoire Vétérinaire Régional de Mostaganem, Algeria. The samples were obtained from different hatcheries and poultry farms as part of routine care and they consisted of broiler and layer breeders, broilers, laying hens, and farm swabs.

### Bacterial strains

After sampling, birds were immediately transported to the laboratory and were aseptically necropsied for the collection of internal organs (liver, pericardium, and spleen). Farm swabs and fecal samples were also used for bacterial isolation. The samples were examined using a previously described method [16], with slight modifications. Briefly, 1 g of sample (internal organs, farm swabs, or fecal samples) was mixed with 9 mL of buffered peptone water (Pasteur Institute of Algeria), vortexed, and incubated overnight at 37°C. To isolate *E. coli*, a drop of broth was streaked on MacConkey agar medium (Biochemica, Spain) and incubated overnight at 37°C. The strains were identified using the analytical profile index 20E system (bioMérieux, Marcy l’Étoile, France) that involves 21 miniaturized biochemical tests.

### Antimicrobial susceptibility testing

The antimicrobial susceptibility testing was done by Kirby–Bauer disk diffusion method in Mueller-Hinton Agar (MHA) using the following disks (Bio-Rad, Marnes-la-Coquette, France): Ampicillin (AM, 10 µg), amoxicillin/clavulanic acid (AMC, 20/10 µg), cephalothin (CEP, 30 μg), XNL (XNL, 30 µg), nalidixic acid (NA, 30 µg), enrofloxacin (ENR, 5 μg), norfloxacin (NX, 10 μg), tetracycline (TE, 30 μg), trimethoprim-sulfamethoxazole (SXT, 1, 25/23, 75 μg), neomycin (N, 30 μg), gentamicin (CN, 10 μg), chloramphenicol (C, 30 μg), nitrofurantoin (FT, 300 μg), and colistin (CT, 50 μg). Antimicrobial susceptibility data were interpreted in compliance with the Clinical and Laboratory Standards Institute (CLSI) guidelines [17,18]. *E. coli* ATCC 25922 was used as a reference strain.

### Screening of MDR and potential ESBL production

In this study, *E. coli* isolates were considered as MDR when resistance to at least one antimicrobial agent of three different families of antibiotics was observed. Screening for ESBL production was done by double-disk synergy test as previously described [19] by filing AMC disk to 30 mm (center to center) of a XNL disk on MHA. The test was considered as positive when a decreased susceptibility to XNL was observed in combination with a champagne cork aspect (a clear-cut enhancement of the inhibition zone of XNL in front of the clavulanate-containing disk). Isolates showing a phenotype of resistance (or reduced susceptibility) to the third-generation cephalosporins without clear synergy were identified as potential ESBL producers, as recommended by CLSI guidelines, which will require further confirmation test.

### Combination disk diffusion ESBL confirmation test

Isolates presumed to be ESBL producers were subjected to a Combination Disk Test (CDT), following CLSI guidelines, using XNL disk alone, and in combination with clavulanic acid, disk applied onto an MHA plate streaked with a 0.5 McFarland bacterial suspension. The inhibition zone diameters were then measured after incubation of 18 h at 37°C. Isolates were considered as ESBL producers when an increase of ≥5 mm in the inhibition zone of the combination disks in comparison to that of the XNL disk alone was observed [18]. *E. coli* ATCC 25922 (American Type Culture Collection, Rockville, MD, USA) was used as a reference strain.

## Results and Discussion

### Antimicrobial resistance

**Figure-1:**
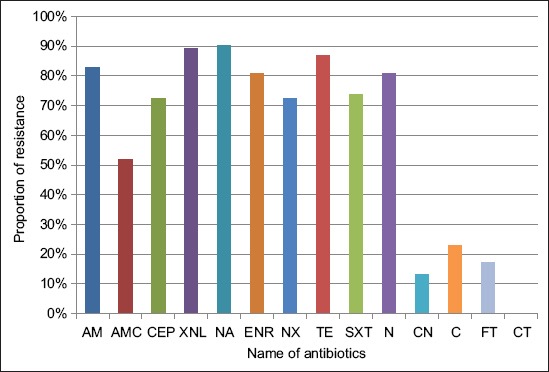
Antimicrobial resistance rates of *Escherichia coli* strains isolated from poultry. TE=Tetracycline, AM=Ampicillin, CEP=Cephalothin, AMC=Amoxicillin/clavulanic acid, XNL=Ceftiofur, N=Neomycin, CN=Gentamicin, SXT=Trimethoprim-sulfamethoxazole, NA=Nalidixic acid, ENR=Enrofloxacin, NX=Norfloxacin, CT=Colistin, FT=Nitrofurantoin, C=chloramphenicol.

From 290 samples received in the Laboratoire Vétérinaire Régional de Mostaganem, Algeria, 145 *E. coli* strains were isolated. All the isolates were screened for susceptibility to a panel of 14 antimicrobials, including commonly used first-line agents. In Algeria, the currently first-line available options for treating severe infections due to *E. coli* in poultry include fluoroquinolones, aminoglycosides, and SXT and in certain cases, penicillins. In this study, antimicrobial susceptibility testing results showed high resistance rates against most of the antibiotics tested. The antibiotic resistance levels are shown in Figure-1. Resistance to NA was most common (90.34%, n=131), followed by TE (86.89%, n=126), AM (82.75%, n=120), ENR (80.68%, n=117) and N (80.68%, n=117), SXT (73.79%, n=107), NX (72.41%, n=105), CEP (72.41%, n=105), AMC (51.72%, n=75), C (22.75%, n=33), FT (17.24%, n=25), and CN (13.10%, n=19) and the lowest resistance level was observed against XNL (3.44%). CT remained effective against all the tested isolates. The resistance rates to the first-line agents tested in this study were higher than those previously reported in Algeria [20,21], as well as in other countries [22,23]. The high resistance rates to currently used first-line antibiotics in our country are troubling. This could be due to their uncontrolled use in poultry, due to the fact that they are administered to whole flocks rather than individual animals, and/or due to the fact that they are prescribed without prior *in vitro* antimicrobial susceptibility testing.

*E. coli* isolates present also a high level of resistance against TE. The high frequency of TE resistance in *E. coli* from chickens agrees with the findings of other studies on antibiotic resistance in *E. coli* [24,25]. TE is a traditional used first-line antibiotic for many domestic animals and the high proportion of TE resistance probably reflects the long history of using this antibiotic for treatment and prophylactic purposes [26]. Resistance to TE is plasmid-mediated, with a wide variety of genetic determinants [27]. This makes it more possible for a susceptible bacterium to acquire resistance factors by conjugation or by transformation as was previously demonstrated [28].

Among *E. coli* isolates, the frequency of resistance to the first-generation cephalosporin CEP occurred at a rate of 72.41%. This molecule is not usually used as a first choice and the high proportion of resistance to this antimicrobial could be the result of coselection phenomenon.

Our study recorded moderate resistance levels to C and FT. These agents are prohibited in food-producing animals in Algeria. Persistence of resistance to these antibiotics has also been reported previously [18], and this might be the result of coselection phenomenon by mobile resistance elements carrying multiple antimicrobial resistance genes [29].

### MDR

**Figure-2:**
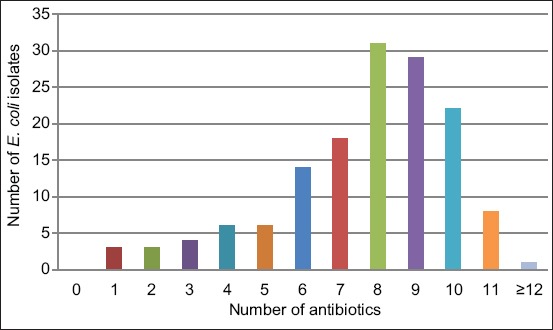
Multidrug-resistant *Escherichia coli* strains isolated from poultry.

All *E. coli* strains isolated in this study were resistant to at least one antibiotic and the majority of them (95.86%, n=139) demonstrated MDR to at least three unrelated antimicrobials. Furthermore, 21.37% were resistant to more than nine antimicrobials. Nine isolates were found to be resistant to more than 10 antibiotics out of 14 antibiotics tested (Figure-2). Most MDR isolates showed resistance to multiple first-line antibiotics that included ENR, SXT, N, and AM as indicated in Table-1. Interestingly, the most common patterns were profiles F, G, and H with a percentage of 14.48%, 12.41%, and 11.03%, respectively. The high frequency of MDR *E. coli* found in the present study might be a result of the common use of combinations of antibiotics and of broad-spectrum antibiotics in the study area, as previously discussed [16].

**Table-1:** The most frequent antibiotic resistance patterns of *Escherichia coli* isolates (n=145).

Resistance patterns	Designation	Number of strains (%)
NA, TE, ENR	A	3 (2.06)
NA, TE, ENR, AM	B	2 (1.37)
NA, TE, ENR, AM, SXT	C	4 (2.75)
NA, TE, ENR, AM, SXT, NX	D	5 (3.44)
NA, TE, ENR, AM, SXT, NX, AMC, N	E	9 (6.20)
NA, TE, ENR, AM, SXT, NX, AMC, CEF	F	21 (14.48)
NA, TE, ENR, AM, SXT, NX, AMC, CEF, N	G	18 (12.41)
NA, TE, ENR, AM, SXT, NX, AMC, CEF, N, C	H	16 (11.03)
NA, TE, ENR, AM, SXT, NX, CEP, N, C, CN	I	4 (2.75)
NA, TE, ENR, AM, SXT, NX, CEP, N, C, CN, FT	J	2 (1.37)
NA, TE, ENR, AM, SXT, NX, CEP, CN, FT, C	K	2 (1.37)
NA, TE, ENR, AM, SXT, NX, CEP, N, C	L	2 (1.37)
Total		88 (60.68)

TE=Tetracycline, AM=Ampicillin, CEP=Cephalothin, AMC=Amoxicillin/clavulanic acid, XNL=Ceftiofur, N=Neomycin, CN=Gentamicin, SXT=Trimethoprim-sulfamethoxazole, NA=Nalidixic acid, ENR=Enrofloxacin, NX=Norfloxacin, FT=Nitrofurantoin, C=Chloramphenicol

### ESBL-producing isolates

Susceptibility testing detected five strains resistant to XNL (3.44%). These isolates were then confirmed by CDT as ESBL producers. ESBL-producing *E. coli* have become a worldwide problem after their first detection in Europe in the 1980s [30]. In the past decade, many studies have alerted about the widespread dissemination of ESBL-producing *E. coli* in food-producing animals [31]. The use of third-generation cephalosporins is uncommon in Algerian poultry production [32]. However, several recent studies have documented the occurrence of cephalosporin-resistant *E. coli* in poultry [33,34]. The presence of ESBL-producing *E. coli* in broiler chickens in our country may be the result of the selection of ESBL-producing *E. coli* clones by an abusive use of other antibiotics (quinolones) in certain poultry sectors and has spread horizontally or by their selection in breeders and their vertical transmission in the poultry production pyramid as previously suggested [35]. Indeed, cephalosporin-resistant *E. coli* have been recently reported from the ovaries of healthy broiler breeders in our country [36].

ESBL-positive *E. coli* isolates demonstrated coresistance against most of the commonly used first-line antibiotics (Table-2). Association with resistance to non-β-lactam antibiotics has also been previously reported in ESBL-positive *E. coli* isolates [34], and this was most often related with plasmid-encoded antibiotic resistance. ESBLs encoding genes in Enterobacteriaceae are often localized on plasmids carrying genes encoding resistance to other families of antibiotics including aminoglycosides and fluoroquinolones [37].

**Table-2:** Multidrug resistance of extended-spectrum β-lactamase-producing *Escherichia coli* isolates.

Antibiotic	S1	S2	S3	S4	S5
AM	R	R	R	R	R
AMC	R	S	R	S	S
CEP	R	R	R	R	R
XNL	R	R	R	R	R
NA	R	R	R	R	R
ENR	R	S	R	R	R
NX	R	S	S	R	R
TE	R	R	R	R	R
SXT	R	S	S	R	R
N	R	S	S	S	S
CN	S	S	S	S	S
C	S	S	S	R	S
FT	S	S	S	R	S
CT	S	S	S	S	S

TE=Tetracycline, AM=Ampicillin, CEP=Cephalothin, AMC=Amoxicillin/clavulanic acid, XNL=Ceftiofur, N=Neomycin, CN=Gentamicin, SXT=Trimethoprim-sulfamethoxazole, NA=Nalidixic acid, ENR=Enrofloxacin, NX=Norfloxacin, CT=Colistin, FT=Nitrofurantoin, C=Chloramphenicol

The results of the present study are of immense concern because some *E. coli* strains are pathogenic and are responsible for a number of serious infections in both human and veterinary medicine. Recent findings describe that resistant organisms or their genes can be transmitted from animals to humans by direct contact, through the food chain, or through environmental contamination [38]. Indeed, it has been suggested that *E. coli* isolates from poultry are genetically related to human pathogenic *E. coli* [39]. Wu *et al*. [40] reported that among animal isolates subjected to multilocus sequence typing (MLST) (n=258), only 1.2% (n=3) were more than 70% similar to human isolates in gene profiles and shared the same MLST clonal complex with the corresponding human isolates.

Based on the results of this study, the first-line antibiotics that are regularly used for empiric therapy of serious infections, such as the fluoroquinolones, are become not effective against MDR *E. coli*. Therefore, the treatment of infections due to these bacteria will remain a challenge to veterinarians because the number of antimicrobial agents reliably effective against the majority of MDR *E. coli* isolated in this study is very limited. This suggests worse clinical outcomes in poultry production, resulting in significant health and welfare concerns and economic losses. Therefore, reasonable use of antibiotics, the adoption of biosecurity measures, and adequate vaccination protocols in poultry production pyramid are becoming mandatory. Furthermore, the valorization of the existing alternatives to antibiotics, including plant extracts [41] and essential oils [42-44], and continuous development of new alternatives to antibiotics are needed to reduce antibiotic usage in food-producing animals.

## Conclusion

The alarming rate of *E. coli* isolates resistant to almost all of the commonly used first-line antibiotics was detected in this study. These results will contribute to describe the updated resistance epidemiological status of *E. coli* in the study area that helps veterinarians to target their treatment choices better. This finding underscores the need to monitor bacteria resistant to the currently used first-line antibiotics in poultry production in other regions in the country as their emergence is an important health concern among most in the food safety community. These results call for taking drastic measures to preserve antibiotic effectiveness and reduce the emergence risks of extensively drug-resistant and pandrug-resistant *E. coli*. Therefore, better prevention of *E. coli* infections and careful choice of antibiotics based on the surveillance programs is necessary to avoid treatment failures and to prevent transmission of antimicrobial residues from poultry production to human food chain. More responsible use of antimicrobials in humans and animals is paramount to make sure antimicrobials remain effective.

## Authors’ Contributions

MBB carried out the main research works and analyzed the main data in the experiments. HA has supervised the laboratory work. QB drafted and revised the manuscript. All authors read and approved the final manuscript.
